# Effect of mask coverage on face identification in Taiwanese men and women

**DOI:** 10.3389/fpsyg.2023.1082376

**Published:** 2023-01-17

**Authors:** Yi-Lang Chen, Cheng-Yu Wu, Shih-Cheng Li, Tai-Min Yu, Shu-Ping Yu

**Affiliations:** Department of Industrial Engineering and Management, Ming Chi University of Technology, New Taipei, Taiwan

**Keywords:** mask coverage, face identification, identification accuracy, sex, identification time

## Abstract

Mask wearing is the easiest and most effective way to avoid COVID-19 infection; however, it affects interpersonal activities, especially face identification. This study examined the effects of three mask coverage levels (full coverage, FC; coverage up to the middle [MB] or bottom of the nose bridge [BB]) on face identification accuracy and time. A total of 115 university students (60 men and 55 women) were recruited to conduct a computer-based simulation test consisting of 30 questions (10 questions [five face images each of men and women] for the three mask coverage levels). One unmasked target face and four face images with a specified mask coverage level were designed for each question, and the participants were requested to select the same face from the four covered face images on the basis of the target face. The ANOVA results indicated that identification accuracy was significantly affected by sex (*p* < 0.01) and the mask coverage level (*p* < 0.001), whereas identification time was only influenced by sex (*p* < 0.05). The multiple comparison results indicated that the identification accuracy rate for faces wearing a mask with FC (90.3%) was significantly lower than for those wearing masks with coverage up to the MB (93.7%) and BB (94.9%) positions; however, no difference in identification accuracy rate was observed between the MB and BB levels. Women exhibited a higher identification accuracy rate than men (94.1% vs. 91.9%) in identifying unfamiliar faces, even though they may spend less time identifying the images. A smaller mask coverage level (i.e., the BB level) does not facilitate face identification. The findings can be served as a reference for people to trade-off between wearing a mask and interpersonal interaction in their daily activities.

## Introduction

Since the outbreak of the COVID-19 pandemic in 2020, more than 500 million people have been infected, resulting in more than 6.3 million deaths worldwide (World Health Organization [WHO], 2022). Mask wearing is the easiest and most effective method to avoid coronavirus infection. The United States Centers for Disease Control and Prevention (CDC, 2020) has suggested that all people should cover their lower face in public settings. Governments around the world have also made similar suggestions, often requiring citizens to wear face masks in public areas or on public transport (Al Jazeera News, 2020). In Taiwan, without exception, people had been stipulated to wear masks in public spaces from December 2020, and the regulations continue to this day (Taiwan Centers for Disease Control [TCDC], 2022). Although the CDC has recommended a standard protocol for wearing masks, however, people alter the coverage of face masks to maximize their comfort level or to facilitate identification by others.

Although masks are effective in inhibiting COVID-19 spread, their implementation has caused changes in people’s lifestyles. People’s facial expressions are covered when using a face mask, thus affects their feelings and cognitions during interpersonal interactions (Cartaud et al., 2020). Wearing masks in public poses a challenge for requiring facial recognition and recognition. A study reported crimes committed by individuals wearing face masks, presumably to disguise or hide their appearance (Southall and Van Syckle, 2020). Faces are often used as a way of verifying identity of an individual, whether across borders or buying alcohol at the local stores, but masks hinder these recognitions (Carragher and Hancock, 2020). Mask wearing also affects interpersonal activities, for example, individuals may have difficulty in identifying known people. Although the identification of people with partially covered or occluded faces is challenging, relatively little research has focused on how covering the lower face influences perceptual face identifications.

Because face masks conceal the lower face (i.e., the mouth and nose), they hinder social interactions and identifications. Recent studies have examined the effects of face masks on face-matching performance (Carragher and Hancock, 2020; Noyes et al., 2021). Although large differences exist among individuals (Estudillo et al., 2021), face masks impair overall face-matching performance (Carragher and Hancock, 2020; Noyes et al., 2021). Factors affecting face identification accuracy have also been explored. For example, an unfamiliar face matching is often error-prone under optimal conditions (Burton et al., 2010; Kramer et al., 2018), and performance deteriorates further in worse conditions (Fysh and Bindemann, 2017). Even minor differences between the images can influence the accuracy, such as color images or black and white images (Bobak et al., 2019), the distances between the individual and the camera in each image (Noyes and Jenkins, 2017), the image quality (Bindemann et al., 2013), the shown viewpoints of the faces (Estudillo and Bindemann, 2014), spectacle usage (Graham and Ritchie, 2019; Noyes et al., 2021), and the lighting conditions (Hill and Bruce, 1996). Moreover, a long time interval between the identification of two images may reduce the accuracy (Megreya et al., 2013). Even if the two pictures are taken just minutes apart, participants make errors in approximately 20% of trials (Burton et al., 2010). Difficulty in unfamiliar face matching was observed for the comparisons of two images (Megreya and Burton, 2006; Burton et al., 2010) and an image with a real person (White et al., 2014). By contrast, Carragher and Hancock (2020) discovered that masks have a large damaging effect on face-matching performance, and the degree of impairment was similar in the identification of both familiar and unfamiliar faces. Noyes et al. (2021) suggested that occlusion impedes face identification accuracy irrespective of a familiar or unfamiliar target. Therefore, masks complicate the face identification process, thereby warranting further exploration.

Most previous behavioral studies concerning sex effect on face identification took use of a learning-test paradigm (McKelvie, 1976; Lewin and Herlitz, 2002; Herlitz and Rehnman, 2008; Megreya et al., 2011). During a typical test, a serial list of unfamiliar faces was displayed for few seconds each, and participants were requested to memorize these faces, as they had to identify them from unfamiliar faces later. In such studies, women superiority in face identification was consistently represented by their higher accuracy compared to men (Megreya et al., 2011; Godard et al., 2013; Hansen et al., 2021; Wong and Estudillo, 2022). Furthermore, Sun et al. (2017) used a modified delayed matching-to-sample task to investigate the time course characteristics of face identification by event-related potential (ERP) for both sexes. This quantitative study using ERP technique also verified that women were more accurate and faster than men on the task. However, the effect of mask coverage on face identification for both sexes was not examined in these investigations. The issue is even more relevant during the COVID-19 pandemic.

**Figure 1:**
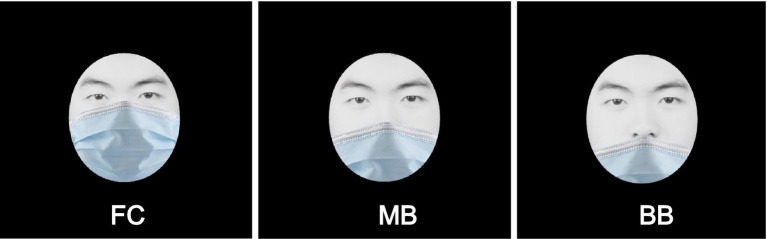
Three common mask coverage levels (FC, full coverage; MB, coverage up to the middle nose bridge; and BB, coverage up to the bottom nose bridge).

Several functions are impeded by wearing masks (e.g., speech, respiration, and comfort; Kumar and Lee, 2020; Rahne et al., 2021; Zhang et al., 2022); therefore, the compound effect of face masks could have a relevant impact on daily life communication even in those with normal respiration. Although the CDC has recommended a standard protocol for wearing masks, we observed that people alter the coverage of face masks (Figure 1) to maximize their comfort level or to facilitate identification by others. According to a survey of Indonesian residents, only 34.3% of the subjects wore face masks properly (Siahaan et al., 2021). Ganczak et al. (2021) further discovered that uncovered noses were the most frequent incorrect practices for masks wearing, with approximately 50% in the country. The COVID-19 pandemic has initiated several studies on face masks; however, no study has evaluated the effect of different mask coverage levels on face identification. Therefore, this study recruited 115 men and women as participants to examine face identification accuracy and time for three mask coverage levels (FC, full coverage; MB, coverage up to the middle nose bridge; and BB, coverage up to the bottom nose bridge; Figure 1). This study hypothesized that a decrease in mask coverage level would increase face identification accuracy.

## Materials and methods

### Participants

A total of 115 university students (60 men and 55 women) with a mean (standard deviation) age of 21.6 (2.8) and 22.8 (5.2) years for men and women, respectively, were recruited. All participants had familiarity with computers (which was required for the test). No participant exhibited self-reported vision defects, such as color blindness or color weakness, which were also checked by an on-line test.^1^ Participants with no vision defects with the naked eye or after vision correction (i.e., wearing glasses) were included. All participants provided informed consent before participating in the study, and the study was approved by the Ethics Committee of Chang Gung Memorial Hospital, Taiwan.

### Stimuli

To examine the effect of mask coverage levels on face identification, three coverage levels (FC, MB, and BB) were adopted. The face images for the test were obtained from the Asian Face Age Dataset (Niu et al., 2016). The screening of these face images was mainly based on the absence of special expressions of emotion to minimize the influence in face identification. We randomly selected 40 frontal male and female face images (20 each); of these, 10 were selected as target images (5 men and 5 women). The images were edited using Adobe Illustrator and Adobe Photoshop 23.5 (Adobe Systems, San Jose, CA, United States) and converted black and white, and the exposure was also standardized. To avoid the image resolution affecting the accuracy of the test, the resolution displayed on a 40-in screen (L42-6500, BenQ, Taipei, Taiwan) was controlled at 1,920 × 1,080 pixels. The plane medical mask (W17.5 cm × H 9.5 cm, Vicvin Tech., Hsinchu, Taiwan) was then superimposed on the faces of the original image to achieve the three coverage levels. A total of 10 questions were developed for each coverage level, and the task involved a target face with a face covering and four choices presented on the subsequent page. In addition to the 10 target images, 30 images were used as non-answer options.

### Experimental design and procedure

Because 10 questions (corresponding to 5 men and 5 women) were developed for each coverage level, the test contained 30 questions for the three levels. A total of 3,450 data sets (115 participants × 3 coverage levels × 10 questions) were recorded, including the participants’ answers and the corresponding identification time. The arrangement of the testing combinations was completely randomized to avoid learning or cumulative bias.

**Figure 2:**
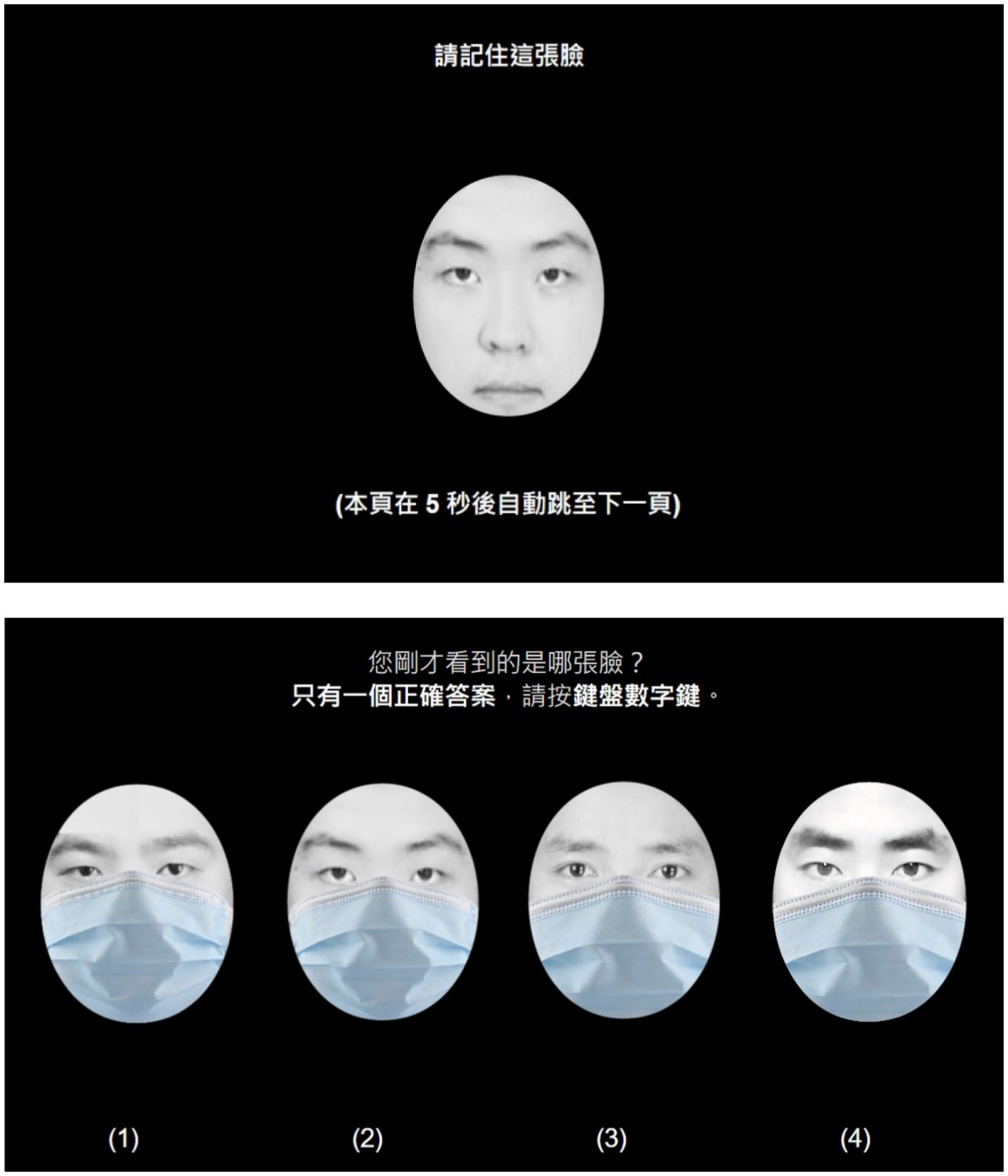
A pilot test format similar to the final test with a full coverage level.

The test was conducted in an isolated quiet room. The participants were requested to sit and follow the instructions displayed on the screen before proceeding to the test. Participants were allowed to adjust the seat height and keyboard position to ensure that their sights were perpendicular to the screen and they were comfortable throughout the experiment. A two-stage practice was conducted before data collection. The first stage was conducted to familiarize the participants with the usage of a computer keyboard and mouse, whereas the second stage was a pilot test with a similar question format to the final test (Figure 2). After that, an alert box was displayed to inform the participants that the practice was complete, and the formal test was going to begin. Upon pressing the OK button, the screen displayed a 8-s countdown to provide time for the participants to answer each question. Once the time has passed and the participant had not answered the question, it was regarded as invalid data, and the screen automatically jumped to the next question. In the test, the participants were requested to key in one correct answer (from four options) for each question until all 30 questions were completed. The total testing time for each participant was approximately 20 min, including instruction, preparation, and formal test.

### Statistical analysis

Statistical analyses were conducted using SPSS 22.0, with significance defined as a minimum α level of 0.05 for all tests. Data collected from the participants were analyzed using descriptive statistics (i.e., means and standard deviations). Two-way analysis of variance (ANOVA) was used to examine the effects of the three coverage levels (FC, MB, and BB) and sex on face identification accuracy and time, and Tukey’s honestly significant difference test was used for multiple comparisons. In addition, one-way ANOVA was conducted to examine the effect of the mask coverage levels on the responses of each sex. A power value was used to examine if the effect size of any significant independent variable was satisfactory (i.e., power ≥ 0.8) as suggested by Cohen (1988).

## Results

### Two-way ANOVA

The two-way ANOVA results of face identification accuracy and time are listed in Tables 1, 2, respectively. Both sex (*p* < 0.01) and mask coverage levels (*p* < 0.001) significantly affected face identification accuracy. Although sex variable affected identification time (*p* < 0.05), the power value was less than 0.8. Women exhibited a higher accuracy rate (94.1% vs. 91.9%) and shorter identification time (2.60 vs. 2.78 s) than men. No interaction was observed in the ANOVA results. Tukey’s test (Table 3) demonstrated that face identification accuracy was lower in the FC level than in the MB and BB levels. No difference in face identification accuracy was observed between the non-FC levels.

**Table 1:** Two-way ANOVA results of face identification accuracy.

Sources	SS	df	MS	*F*	value of *p*	Power
Sex	0.042	1	0.042	8.535	<0.01	0.829
Mask coverage	0.129	2	0.065	13.270	<0.001	0.998
Sex × mask coverage	0.006	2	0.003	0.592	0.554	0.149

**Table 2:** Two-way ANOVA results of identification time.

Sources	SS	df	MS	*F*	value of *p*	Power
Sex	2.606	1	2.606	4.752	<0.05	0.585
Mask coverage	2.656	2	1.328	2.422	0.090	0.487
Sex × mask coverage	0.114	2	0.057	0.104	0.902	0.066

**Table 3:** Tukey’s test results of identification accuracy and time.

Sources	Accuracy (%)	Tukey test	Time (s)	Tukey test
Full coverage (FC)	90.3 (8.4)	A	2.82 (0.75)	A
Middle of nose bridge (MB)	93.7 (6.7)	B	2.63 (0.70)	A
Bottom of nose bridge (BB)	94.9 (5.0)	B	2.63 (0.78)	A

Data (mean with standard deviation in parentheses) with the same letter do not differ in Tukey’s test.

### One-way ANOVA results for men and women

**Figure 3:**
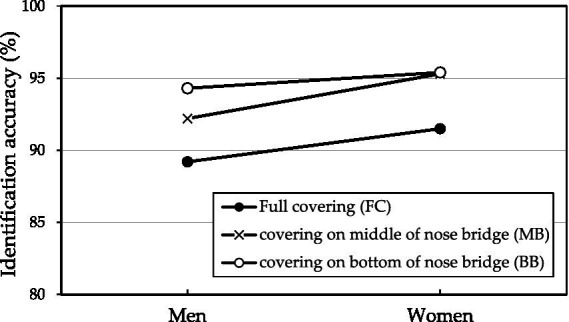
Face identification accuracies of six combinations formed using sex and coverage levels.

The effects of mask coverage levels on the two responses of men and women as analyzed using one-way ANOVA are listed in Table 4. The mask coverage levels significantly affected face identification accuracy but not identification time, regardless of sex. However, men exhibited a significant difference in face identification accuracy between the FC and BB levels. Women exhibited no difference in face identification accuracy between the MB and BB levels, with accuracy in these levels being different from that in the FC level. Figure 3 illustrates the six combinations formed using sex and coverage variables. The trend of face identification accuracy in the MB level is different between sexes.

**Table 4:** One-way ANOVA results of the effects of coverage levels on identification accuracy and time for each sex.

Sex	Response	SS	df	MS	*F*	value of *p*	Power
Men	Accuracy	0.081	2	0.040	6.573	<0.01	0.906
	Time	0.935	2	0.468	0.754	0.472	0.177
Women	Accuracy	0.055	2	0.028	7.992	<0.001	0.953
	Time	1.797	2	0.898	1.911	0.151	0.151

## Discussion

Although studies have indicated that mask wearing affects face identification accuracy (Carragher and Hancock, 2020; Noyes et al., 2021), no study has evaluated the effects of different mask coverage levels on face identification accuracy. On the basis of field observations, this study adopted three commonly used mask-wearing methods (each representing different exposed areas of the face) and examined their effects on identification accuracy and time. The results verified the hypothesis by demonstrating that less coverage of mask (e.g., non-FC levels) increased face identification accuracy. However, identification accuracy did not decrease from the MB level to the BB level, indicating that face identification accuracy cannot be further improved by putting the mask down to the BB level. However, identification accuracy can be significantly enhanced by putting the mask down from the FC level to the MB level. This implies that the nose may be one of the crucial clues for face identification, even exposing the upper part of nose can also increase the face identification performance.

Although face identification accuracies in this study were higher than those in previous studies, a large error variance in face matching can be explained by the face stimulus characteristics, indicating that specific stimulus features might enhance the identification of masked faces (Estudillo and Bindemann, 2014; Fysh and Bindemann, 2017). Experiments of face identification involved the use of different materials, recognizer characteristics [e.g., super recognizers, Noyes et al., 2021], and testing protocols; therefore, direct comparisons among studies are challenging. The commonly used identification methods in past research included matching (Jenkins et al., 2011; Carragher and Hancock, 2020), recalling (Davies et al., 1977; Burton et al., 2010), image comparison (Megreya and Burton, 2006; Burton et al., 2010), the corresponding target face identified from options (Duchaine and Nakayama, 2006), and shelters (Nguyen and Pezdek, 2017; Noyes et al., 2021; Estudillo and Wong, 2022). Behavioral and neuropsychological studies have also demonstrated that cognitive processes among different approaches reflect distinct cognitive mechanisms (Bindemann and Burton, 2021). Megreya and Burton (2007) also revealed some dissociations between identity match and mismatch trials.

Investigations have determined that matching unfamiliar faces is more challenging than matching familiar ones (Megreya and Burton, 2006). Although this study employed unfamiliar faces as testing subjects, identification accuracy rates were all higher than nearly 90%. This may be because identification of the upper face (Fisher and Cox, 1975; Davies et al., 1977; Dal Martello and Maloney, 2006), specifically the eyes (McKelvie, 1976; Roberts and Bruce, 1988), is more accurate than that of the lower face (e.g., the nose, mouth, and chin). Studies have determined that the eyes are the most crucial cue for face identification. Fisher and Cox (1975) and McKelvie (1976) observed that the identification of faces with covered eyes was less accurate than that of faces with a covered mouth. Roberts and Bruce (1988) also suggested that covered eyes caused a more significant impact on face identification. Recent studies suggested that covering the face inhibited the recognition of identity and emotional expressions. However, it may also make the eyes more prominent, since they are a reliable index to orient people’s social and spatial attention (Villani et al., 2022). In addition, a questionnaire with a forced 4-choice-1-question format was used in this study, and the participants tended to choose the closest answer. Although the participants were not necessarily certain, they recognized that the correct answer existed among the four options, which may also improve identification accuracy.

Identification accuracy and time differed between men and women. A study on female facial expression identification demonstrated that women have faster and more accurate reaction times than men (Lewin and Herlitz, 2002), and also confirmed by other studies (Megreya et al., 2011; Godard et al., 2013; Hansen et al., 2021; Wong and Estudillo, 2022). Lewin and Herlitz (2002) indicated that women’s higher face recognition performance was hypothesized to be related to either their higher verbal ability or to their superiority in recognizing female faces. Another face processing study reported that women were better at face recognition than men because they may make more fixations in short fixation durations than men (Herlitz and Rehnman, 2008). Women were faster and more accurate in female facial expression recognition than men, and women looked more at the eyes than men. That is, the superior performance of women in facial expression recognition is related to greater female attention to the eyes, whereas men focus more on the mouth (Hall et al., 2010). The occlusion of the mouth by masks may be a reason for the lower identification accuracy and longer identification time in men than in women. Therefore, the trend of identification accuracy in the BB level differed between men and women (Table 5; Figure 3). This implies that different facial exposure cues caused varying accuracy rates between men and women, therefore, the eye-tracking technique may be a useful tool to clarify the study result in future investigation.

**Table 5:** Tukey’s test results of the effects of coverage levels on identification accuracy and time for each sex.

Men (*N* = 60)		Women (*N* = 55)		
Coverage	Accuracy (%)	Time (s)	Accuracy (%)	Time (s)
FC	89.2 (8.7) A	2.88 (0.73) A	91.5 (6.9) A	2.75 (0.76) A
MB	92.2 (8.2) AB	2.71 (0.74) A	95.3 (5.2) B	2.53 (0.64) A
BB	94.3 (5.3) B	2.75 (0.88) A	95.5 (5.3) B	2.53 (0.65) A

Data (mean with standard deviation in parentheses) with the same letter do not differ in the Tukey’s test; FC, full coverage; MB, coverage up to the middle nose bridge; BB, coverage up to the bottom nose bridge.

This study has several limitations. Although 115 participants were recruited, the power value of the ANOVA result (sex effect on identification time, Table 2) was less than 0.8, implying the insufficiency of the sample size. This may affect the generalization of the study results. In addition, the overall identification accuracy rates were higher than those in previous studies, which may be because of the forced 4-choice-1-question questionnaire format. An additional option of “no corresponding face” among the candidate answers may have reduced the possibility of the correct option surmised by the participants, thereby bringing the identification accuracy rate closer to the fact. Furthermore, to control for other interfering variables, we removed the features other than the face used in the test, such as hair, ears, and face shape, resulting in an image that may be slightly different from the actual one. Once other facial features were also appeared, the face may become easier to be identified and needs further verification.

## Conclusion

The emergence of the COVID-19 pandemic has led to changes in human communications and interactions. Face masks are commonly used in public spaces. This study examined the effects of three mask coverage levels (FC, MB, and BB) on face identification accuracy and time. The results indicated that identification accuracy and time were significantly affected by sex, and identification accuracy was also influenced by the mask coverage level. During face identification, identification accuracy cannot be further improved by putting the mask at the BB level. Moreover, we recommend wearing masks at the MB level during face identification.

## Data availability statement

The raw data supporting the conclusions of this article will be made available by the authors, without undue reservation.

## Ethics statement

The studies involving human participants were reviewed and approved by the Ethics Committee of Chang Gung Memorial Hospital, Taiwan. Participants provided written informed consent to participate in this study. Written informed consent was obtained from the individual(s) for the publication of any potentially identifiable images or data included in this article.

## Author contributions

All authors listed have made a substantial, direct, and intellectual contribution to the work and approved it for publication.

## Funding

This work was supported by the National Science and Technology Council (NSTC), Taiwan (#110-2221-E-131-025-MY3).

## Conflict of interest

The authors declare that the research was conducted in the absence of any commercial or financial relationships that could be construed as a potential conflict of interest.

## Publisher’s note

All claims expressed in this article are solely those of the authors and do not necessarily represent those of their affiliated organizations, or those of the publisher, the editors and the reviewers. Any product that may be evaluated in this article, or claim that may be made by its manufacturer, is not guaranteed or endorsed by the publisher.

## Footnotes

^1^ https://www.ifreesite.com/color-blindness

## References

[ref1] Al Jazeera News (2020). Which countries have made wearing face masks compulsory? Available at: https://www.aljazeera.com/news/2020/04/countries-wearing-face-masks-compulsory-200423094510867.html

[ref2] Bindemann M. Attard J. Leach A. Johnston R. A. (2013). The effect of image pixelation on unfamiliar-face matching. Appl. Cogn. Psychol. 27, 707–717. doi: 10.1002/acp.2970

[ref3] Bindemann M. Burton M. (2021). “Steps towards a cognitive theory of unfamiliar face matching,” in Forensic Face Matching: Research and Practice. ed. Bindemann M. (Oxford, England: Oxford University Press), 38–61.

[ref4] Bobak A. K. Mileva V. R. Hancock P. J. (2019). A grey area: how does image hue affect unfamiliar face matching? Cogn. Res. Princ. Implic. 4:27. doi: 10.1186/s41235-019-0174-3 31332556 PMC6646495

[ref5] Burton A. M. White D. McNeill A. (2010). The Glasgow face matching test. Behav. Res. Methods 42, 286–291. doi: 10.3758/BRM.42.1.286 20160307

[ref6] Carragher D. J. Hancock P. J. (2020). Surgical face masks impair human face matching performance for familiar and unfamiliar faces. Cogn. Res. Princ. Implic. 5:59. doi: 10.1186/s41235-020-00258-x, PMID: 33210257 PMC7673975

[ref7] Cartaud A. Ott L. Iachini T. Honoré J. Coello Y. (2020). The influence of facial expression at perceptual threshold on electrodermal activity and social comfort distance. Psychophysiology 57:e13600. doi: 10.1111/psyp.13600 32437046

[ref8] CDC (2020). Recommendation Regarding the Use of Cloth Face Coverings, Centers for Disease Control and Prevention, Atlanta, GA.

[ref9] Cohen J. (1988). Statistical Power Analysis for the Behavioral Sciences, 2nd Edn. Erlbaum, Hillsdale, NJ.

[ref10] Dal Martello M. F. Maloney L. T. (2006). Where are kin recognition signals in the human face? J. Vis. 6, 1356–1366. doi: 10.1167/6.12.2 17209739

[ref11] Davies G. Ellis H. Shepherd J. (1977). Cue saliency in faces as assessed by the ‘Photoft’technique. Perception 6, 263–269. doi: 10.1068/p060263, PMID: 866082

[ref12] Duchaine B. Nakayama K. (2006). The Cambridge face memory test: results for neurologically intact individuals and an investigation of its validity using inverted face stimuli and prosopagnosic participants. Neuropsychologia 44, 576–585. doi: 10.1016/j.neuropsychologia.2005.07.001, PMID: 16169565

[ref13] Estudillo A. J. Bindemann M. (2014). Generalization across view in face memory and face matching. Iperception 5, 589–601. doi: 10.1068/i0669, PMID: 25926967 PMC4411982

[ref14] Estudillo A. J. Hills P. Wong H. K. (2021). The effect of face masks on forensic face matching: an individual differences study. J. Appl. Res. Mem. Cogn. 10, 554–563. doi: 10.1037/h0101864

[ref15] Estudillo A. J. Wong H. K. (2022). Two face masks are better than one: congruency effects in face matching. Cogn. Res. Princ. Implic. 7:49. doi: 10.1186/s41235-022-00402-9, PMID: 35674914 PMC9175166

[ref16] Fisher G. Cox R. (1975). Recognizing human faces. Appl. Ergon. 6, 104–109. doi: 10.1016/0003-6870(75)90303-8 15677175

[ref17] Fysh M. C. Bindemann M. (2017). “Forensic face matching: a review” in Face Processing: Systems, Disorders and Cultural Differences. eds. Bindemann M. Megreya A. M. (Hauppauge, NY: Nova Science Publishers), 1–20.

[ref18] Ganczak M. Pasek O. Duda-Duma Ł. Świstara D. Korzeń M. (2021). Use of masks in public places in Poland during SARS-CoV-2 epidemic: a covert observational study. BMC Public Health 21:393. doi: 10.1186/s12889-021-10418-3 33622279 PMC7901005

[ref19] Godard O. Baudouin J. Y. Bonnet P. Fiori N. (2013). Identity–expression interaction in face perception: sex, visual field, and psychophysical factors. Laterality 18, 594–611. doi: 10.1080/1357650X.2012.734312, PMID: 23163487

[ref20] Graham D. L. Ritchie K. L. (2019). Making a spectacle of yourself: the effect of glasses and sunglasses on face perception. Perception 48, 461–470. doi: 10.1177/0301006619844680, PMID: 31006340

[ref21] Hall J. K. Hutton S. B. Morgan M. J. (2010). Sex differences in scanning faces: does attention to the eyes explain female superiority in facial expression recognition? Cogn. Emot. 24, 629–637. doi: 10.1080/02699930902906882

[ref22] Hansen T. Zaichkowsky J. de Jong A. (2021). Are women always better able to recognize faces? The unveiling role of exposure time. PLoS One 16:e0257741. doi: 10.1371/journal.pone.0257741, PMID: 34710131 PMC8553055

[ref23] Herlitz A. Rehnman J. (2008). Sex differences in episodic memory. Curr. Dir. Psychol. Sci. 17, 52–56. doi: 10.1111/j.1467-8721.2008.00547.x

[ref24] Hill H. Bruce V. (1996). The effects of lighting on the perception of facial surfaces. J. Exp. Psychol. Hum. Percept. Perform. 22, 986–1004. doi: 10.1037/0096-1523.22.4.986 8756964

[ref25] Jenkins R. White D. Van Montfort X. Burton M. (2011). Variability in photos of the same face. Cognition 121, 313–323. doi: 10.1016/j.cognition.2011.08.001, PMID: 21890124

[ref26] Kramer R. S. Young A. W. Burton A. M. (2018). Understanding face familiarity. Cognition 172, 46–58. doi: 10.1016/j.cognition.2017.12.005 29232594

[ref27] Kumar S. Lee H. P. (2020). The perspective of fluid flow behavior of respiratory droplets and aerosols through the facemasks in context of SARS-CoV-2. Phys. Fluids 32:111301. doi: 10.1063/5.0029767, PMID: 33281434 PMC7713871

[ref28] Lewin C. Herlitz A. (2002). Sex differences in face recognition—Women’s faces make the difference. Brain Cogn. 50, 121–128. doi: 10.1016/S0278-2626(02)00016-7 12372357

[ref29] McKelvie S. J. (1976). The role of eyes and mouth in the memory of a face. Am. J. Psychol. 89, 311–323. doi: 10.2307/1421414

[ref30] Megreya A. M. Bindemann M. Havard C. (2011). Sex differences in unfamiliar face identification: evidence from matching tasks. Acta Psychol. 137, 83–89. doi: 10.1016/j.actpsy.2011.03.003, PMID: 21459354

[ref31] Megreya A. M. Burton A. M. (2006). Unfamiliar faces are not faces: evidence from a matching task. Memory Cogn. 34, 865–876. doi: 10.3758/BF03193433 17063917

[ref32] Megreya A. M. Burton M. A. (2007). Hits and false positives in face matching: a familiarity-based dissociation. Percept. Psychophys. 69, 1175–1184. doi: 10.3758/BF03193954, PMID: 18038955

[ref33] Megreya A. M. Sandford A. Burton A. M. (2013). Matching face images taken on the same day or months apart: the limitations of photo ID. Appl. Cogn. Psychol. 27, 700–706. doi: 10.1002/acp.2965

[ref34] Nguyen T. B. Pezdek K. (2017). Memory for disguised same-and cross-race faces: the eyes have it. Visual Cogn. 25, 762–769. doi: 10.1080/13506285.2017.1329762

[ref35] Niu Z. Zhou M. Wang L. Gao X. Hua G. (2016). Ordinal regression with multiple output CNN for age estimation. In Proceedings of the IEEE Conference on Computer Vision and Pattern Recognition, Las Vegas, NV. 4920–4928.

[ref36] Noyes E. Davis J. P. Petrov N. Gray K. L. Ritchie K. L. (2021). The effect of face masks and sunglasses on identity and expression recognition with super-recognizers and typical observers. R. Soc. Open Sci. 8:201169. doi: 10.1098/rsos.201169 33959312 PMC8074904

[ref37] Noyes E. Jenkins R. (2017). Camera-to-subject distance affects face configuration and perceived identity. Cognition 165, 97–104. doi: 10.1016/j.cognition.2017.05.012, PMID: 28527319

[ref38] Rahne T. Fröhlich L. Plontke S. Wagner L. (2021). Influence of surgical and N95 face masks on speech perception and listening effort in noise. PLoS One 16:e0253874. doi: 10.1371/journal.pone.0253874, PMID: 34197513 PMC8248731

[ref39] Roberts T. Bruce V. (1988). Feature saliency in judging the sex and familiarity of faces. Perception 17, 475–481. doi: 10.1068/p170475, PMID: 3244520

[ref40] Siahaan A. M. P. Lubis M. P. Dalimunthe D. A. Nasution M. R. Lubis H. P. R. (2021). Adherence to face mask and social distancing among residents in Medan during the COVID-19 pandemics. Bali Med. J. 10, 529–533. doi: 10.15562/bmj.v10i2.2414

[ref41] Southall A. Van Syckle K. (2020). Coronavirus bandits? 2 armed men in surgical masks rob racetrack. The New York Times. Available at: https://www.nytimes.com/2020/03/08/nyregion/aqueduct-racetrack-robbery.html

[ref42] Sun T. Li L. Xu Y. Zheng L. Zhang W. Zhou F. A.. (2017). Electrophysiological evidence for women superiority on unfamiliar face processing. Neurosci. Res. 115, 44–53. doi: 10.1016/j.neures.2016.10.002, PMID: 27794442

[ref43] Taiwan Centers for Disease Control [TCDC] (2022). Acts and Regulations. Available at: https://www.cdc.gov.tw/En/Category/Page/yZOu-4cGeu77HDyzE0ojqg#

[ref44] Villani C. D’Ascenzo S. Scerrati E. Ricciardelli P. Nicoletti R. Lugli L. (2022). Wearing the face mask affects our social attention over space. Front. Psychol. 13:923558. doi: 10.3389/fpsyg.2022.923558, PMID: 35992481 PMC9386249

[ref45] White D. Kemp R. I. Jenkins R. Burton A. M. (2014). Feedback training for facial image comparison. Psychon. Bull. Rev. 21, 100–106. doi: 10.3758/s13423-013-0475-3, PMID: 23835616

[ref46] Wong H. K. Estudillo A. J. (2022). Face masks affect emotion categorisation, age estimation, recognition, and gender classification from faces. Cogn. Res.: Princ. Implic 7:91. doi: 10.1186/s41235-022-00438-x, PMID: 36209185 PMC9547636

[ref47] World Health Organization [WHO] (2022). World Health Statistics. Available at: https://www.who.int/data

[ref48] Zhang T. T. Zhang T. Liu S. (2022). A modified surgical face mask to improve protection and wearing comfort. Buildings 12:663. doi: 10.3390/buildings12050663

